# Case report and systematic review of cerebellar vermis alterations in psychosis

**DOI:** 10.1097/YIC.0000000000000535

**Published:** 2024-02-13

**Authors:** Nicola Dusi, Cecilia Maria Esposito, Giuseppe Delvecchio, Cecilia Prunas, Paolo Brambilla

**Affiliations:** a Department of Neurosciences and Mental Health, Fondazione IRCCS Ca’ Granda, Ospedale Maggiore Policlinico, Milan; b Department of Brain and Behavioral Sciences, University of Pavia, Pavia; c Department of Pathophisiology and Transplantation, University of Milan, Milan, Italy

**Keywords:** bipolar disorder, cerebellum, neuroimaging, psychosis, schizophrenia

## Abstract

**Introduction:**

Cerebellar alterations, including both volumetric changes in the cerebellar vermis and dysfunctions of the corticocerebellar connections, have been documented in psychotic disorders. Starting from the clinical observation of a bipolar patient with cerebellar hypoplasia, the purpose of this review is to summarize the data in the literature about the association between hypoplasia of the cerebellar vermis and psychotic disorders [schizophrenia (SCZ) and bipolar disorder (BD)].

**Methods:**

A bibliographic search on *PubMed* has been conducted, and 18 articles were finally included in the review: five used patients with BD, 12 patients with SCZ and one subject at psychotic risk.

**Results:**

For SCZ patients and subjects at psychotic risk, the results of most of the reviewed studies seem to suggest a gray matter volume reduction coupled with an increase in white matter volumes in the cerebellar vermis, compared to healthy controls. Instead, the results of the studies on BD patients are more heterogeneous with evidence showing a reduction, no difference or even an increase in cerebellar vermis volume compared to healthy controls.

**Conclusions:**

From the results of the reviewed studies, a possible correlation emerged between cerebellar vermis hypoplasia and psychotic disorders, especially SCZ, ultimately supporting the hypothesis of psychotic disorders as neurodevelopmental disorders.

## Introduction

Neuroimaging applications offer an important tool of investigation in the research of psychiatric disorders, and, despite the limitations related to the reproducibility of techniques and samples, they are providing more and more useful insight for clinical practice (Anderson *et al*., 2019). So far, various alterations of the central nervous system have been described, both at a morphological and functional level, especially in the field of major psychosis (Voineskos *et al*., 2020). In this context, several biomarkers have been hypothesized to improve the diagnostic and prognostic definition of these patients, with promising results (Aydin *et al*., 2019; Keshavan *et al*., 2020).

At the brain level, morphological and connectivity alterations were frequently found in psychotic subjects (O’Donoghue *et al*., 2015). In particular, many studies suggested the presence of gray matter and white matter reductions in patients with schizophrenia (SCZ), especially in prefrontal regions, thalamus, cingulate cortex, temporal lobe, basal ganglia, amygdala and hippocampus, also coupled with total ventricular volume increase (Brambilla *et al*., 2013; Delvecchio *et al*., 2017; Aydin *et al*., 2019). Moreover, the ability to discriminate patients with SCZ from healthy controls (HC) was also highlighted by a meta-analysis of multivariate pattern recognition studies of neuroimaging-based diagnostic biomarkers, which found an overall sensitivity and specificity of approximately 80% (Kambeitz *et al*., 2015; Kraguljac *et al*., 2021). It is important to underline that brain alterations seem more consistent in psychotic patients with a chronic course than in those with a first psychotic episode, indicating an increase in the burden of the disease over time (Hulshoff Pol and Kahn, 2008; Kambeitz *et al*., 2015). Interestingly, the same alterations have been found in ultra-high-risk (UHR) subjects, therefore suggesting the ability of specific biomarkers to predict the risk of evolution to psychosis in UHR subjects (Koutsouleris *et al*., 2015; Del Fabro *et al*., 2021; Kraguljac *et al*., 2021). As for bipolar disorder (BD), it has been shown that patients with BD have structural alterations in several cortical regions, including prefrontal, anterior temporal and insula cortices (Ganzola and Duchesne, 2017; Dusi *et al.*, (2019); Prunas *et al.*, (2018); Brambilla *et al.*, (2009); Ching *et al*., 2022), and subcortical areas, such as hippocampus, thalamus and amygdala (Hajek *et al*., 2009; Rimol *et al*., 2010), as well as functional connectivity deficits, especially within and between cortico-limbic structures (Cattarinussi *et al*., 2022), compared to HC.

Moreover, when SCZ patients and BD patients undergo a direct comparison, SCZ patients present more extensive gray matter alterations than BD patients, especially in the frontotemporal cortex, thalamus, hippocampus and amygdala (Maggioni *et al*., 2016). In contrast, it has been suggested that BD patients showed a greater reduction in gray matter volumes of the cerebellum compared to SCZ patients (Farrow *et al*., 2005; Molina *et al*., 2011; Maggioni *et al*., 2016). This evidence is not surprising, especially because the cerebellum, and in particular the cerebellar vermis, is considered an ‘emotional pacemaker’ specifically engaged in the modulation of emotional processing and therefore particularly involved in affective disorders (Heath, 1977; Minichino *et al*., 2014). In general, the cerebellar alterations have been extensively described to be related to the pathophysiology of other psychiatric disorders, including attention deficit hyperactivity disorder, autism spectrum disorders, SCZ, major depressive disorder and anxiety disorders (Phillips *et al*., 2015; Laidi *et al*., 2019). The cerebellum has multiple control functions, especially those related to motor coordination, cognitive abilities and emotion regulation (Koziol *et al*., 2014; Mariën and Borgatti, 2018). Although it is not yet certain which function is particularly involved in the pathogenesis of psychiatric disorders, it seems reasonable to support Minichino’s hypothesis, which underlines the centrality of this structure in cognitive and emotional functions, abilities that are often impaired in these conditions (Minichino *et al*., 2014).

The idea of this article arises from the observation of a clinical case, reported below, of a female patient with BD in which hypoplasia of the cerebellar vermis was found to be associated with functional glucose hypo-uptake. The purpose of this article is therefore to review the literature to highlight the correlations described between hypoplasia of the cerebellar vermis and psychotic disorders, in particular UHR, SCZ and BD, to allow an evaluation in diagnostic and prognostic terms.

## Methods

The review was designed according to the PRISMA model for systematic reviews (Page *et al*., 2021). To identify potentially relevant records, a bibliographic search was conducted in the *PubMed* database without any prespecified restriction. To review all scientific articles exploring the association between alterations in the cerebellar vermis and psychosis, we carried out a comprehensive search, where the keywords ‘vermis’, ‘vermal’, ‘medial’, ‘paravermis’, ‘paravermal’, ‘paramedial’ were matched with the terms ‘cerebellar’, ‘cerebellum’, and with the terms ‘psychotic disorder’, ‘psychosis’, ‘schizophrenia’, and ‘bipolar disorder’.

**Fig. 1:**
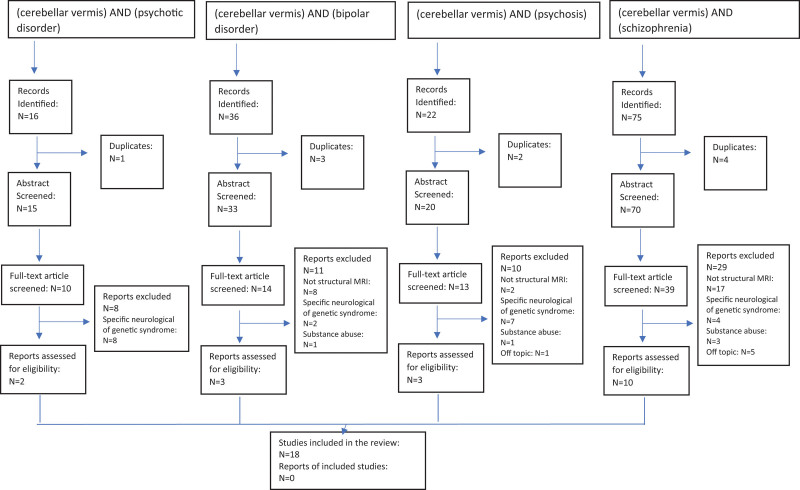
PRISMA diagram for systematic reviews.

Eligibility criteria consisted of (1) articles written in English (or with an English translation available), (2) original studies on human samples: review articles and case reports were excluded, (3) patients with a diagnosis of SCZ or BD and (4) neuroimaging studies using structural MRI. Studies using other neuroimaging methods or samples with comorbid substance abuse and patients with specific neurological or genetic syndromes were excluded to reduce the heterogeneity across the reviewed studies. Records were also considered for inclusion if quoted in the reference lists of articles screened for inclusion. All the resulting records were screened by title and abstract by CME and then full-text articles were evaluated by CME and ND. Briefly, starting from 149 articles screened, 18 articles were finally included in the review. The selection of the articles has been reported in Fig. 1.

Characteristics of the reviewed articles have been summarized in Table 1. For each article, we extracted the following variables: general information (author, year of publication), study design, sample characteristics (demographics and numerosity), diagnosis of the sample (BD, SCZ or UHR), methods (structural MRI with 1.5 Tesla or 3 Tesla), and main results.

**Table 1:** Cerebellar vermis alterations in psychosis

Study	Design	Sample	Diagnosis	Methods	Results
Brambilla *et al*., 2001	Cross-sectional study	22 BD patients (40.91% female, mean age 36 ± 10 years) 22 HC (36.36% female, mean age 38 ± 10 years)	BD	MRI (1.5-T) Scion Image Software	BD vs HC: No significant differences for any posterior fossa measures. Familial patients had smaller left and right cerebellar hemispheres and total vermis volumes, and larger left lateral ventricle volumes compared with nonfamilial ones.
Ichimiya *et al*., 2001	Cross-sectional study	20 drug-naïve SCZ patients (0% female, mean age 28.3 ± 6.9 years) 20 HC (0% female, mean age 26.3 ± 1.8 years)	SCZ	MRI (1.5-T) NIHImage software	SCZ vs HC: Significantly smaller volumes of the vermis, but not of other cerebellar structures. Correlation between reduction in the vermal volume and total Brief Psychiatric Rating Scale Depression subscore and Paranoia subscore.
Loeber *et al*., 2001	Cross-sectional study	38 SCZ patients (0% females, mean age 33.6 ± 12.1 years) 26 HC (0% females, mean age 33.5 ± 11.0 years)	SCZ	MRI (1.5-T) Manual volumetry	SCZ vs. HC: Smaller volumes of the whore vermis, but not of the cerebellar hemispheres: statistical significance only in case of patients with a comorbid diagnosis of alcohol abuse. SCZ without vs. SCZ with alcohol abuse: Similar anomalies of the posterior vermian areas (lobules VI and VII) Abnormalities of the anterior vermis (lobules I–V) were observed only among patients with a dual diagnosis of alcoholism. No difference emerged at the inferior vermian level (lobules VIII–X).
Okugawa *et al*., 2003	Cross-sectional study	59 chronic SCZ patients (35.59% female, male mean age 39.6 ± 7.0 years, female mean age 39.7 ± 7.0 years) 57 HC (35.60% female, male mean age 38.7 ± 7.5 years, female mean age 37.2 ± 8.7 years)	SCZ	MRI (1.5-T) BRAINS software	SCZ vs HC: Significantly smaller total vermis volume and smaller vermian subregions, regardless of gender. No differences in total intracranial volume and cerebellar hemisphere volumes.
Joyal *et al*., 2004	Cross-sectional study	19 SCZ patients (21.05% females, mean age 36 ± 6 years) 19 HC (21.05% females, mean age 27 ± 6 years)	SCZ	MRI (1.5-T) Magic View 1000 software	SCZ vs. HC: Significantly smaller inferior vermis. Significantly smaller cerebellar asymmetry.
Lawyer *et al*., 2009	Cross-sectional study	71 SCZ patients (25.56% female, mean age 40.8 ± 7.6 years) 65 HC (40% female, mean age 44.1 ± 7.7 years)	SCZ	MRI (1.5.T) BRAINS software	SCZ vs HC: Enlarged ventricles, reduced posterior superior vermis GM volume, and increased putamen GM volume. Vermis regions were associated with vigilance, executive function, and, less strongly, visuomotor speed.
Lee *et al*., 2007	Cross-sectional study	40 SCZ patients (0% female, mean age 31.9 ± 9 years) 40 HC (0% female, mean age 31.7 ± 8.9 years)	SCZ	MRI (1.5-T) WFP_PickAtlas v1.04 software, SPM2	SCZ vs HC: Significantly increased cerebellar vermis WM volume. No differences in total cerebellar volume, and GM and WM volumes of cerebellar hemispheres. Increased vermis WM volume in patients was associated with poor verbal fluency performance.
Monkul *et al*., 2008	Cross-sectional study	16 BD patients (50% female, mean age 15.5 ± 3.4 years) 21 HC (42.86% female, mean age 16.9 ± 3.8 years)	BD	MRI (1.5-T) Scion Image software	BD vs HC: No significant differences in cerebellum or vermis measures; however, there was a trend to smaller vermis V2 areas in patients. Inverse correlation between between number of previous affective episodes and vermis area V2 in the male bipolar patient group.
Lawyer *et al*., 2009	Cross-sectional study	52 chronic SCZ or schizoaffective patients (36.54% female, mean age 42 ± 7.0 years) 55 HC (32.73% female, mean age 37 ± 8.8 years)	SCZ	MRI (1.5-T) BRAINS software	SCZ vs HC: Increased mean of GM/WM ratios; trend level of significantly increase of the variances. Disturbed interrelation of tissue class volumes in the cerebellar vermis.
Womer *et al*., 2009	Cross-sectional study	44 BD patients (56.82% female, aged 14–55 years) 43 HC (47.27% female, aged 18–53 years)	BD	MRI (3-T) BioImage Suite software, SPM99	BD vs HC: Significantly larger total vermis volumes in males with BD, not in females with BD. Significantly larger subregion V1 volumes in BD, with differences primarily driven by males.
Henze *et al*., 2011	Cross-sectional study	13 adolescent SCZ patients 13 HC age- and gender-matched	SCZ	MRI (1.5-T) SPM5	SCZ vs HC: Decreased GM in the cerebellar vermis, and alterations in the left putamen.
Rasser *et al*., 2010	Cross-sectional study	13 remitted first-episode SCZ patients with less than 2 years’ duration of illness (7.69% female, mean age 20.7 ± 3.6 years) 13 HC (7.69% female, mean age 21.6 ± 2.8 years)	SCZ	MRI (1.5-T) Manual volumetry	SCZ vs HC: No differences in total cerebellar volume and total GM volume. Increased total cerebellar WM and reduction of total GM/WM ratios. Clusters of cerebellar GM reduction identified: in superior vermis; in the left lobuli VI; in right-inferior lobule IX, extending into left lobule IX; and bilaterally in the areas of lobuli III, peduncle and left flocculus.
Baldaçara *et al*., 2011	Cross-sectional study	40 euthymic BD patients (72.50% female, mean age 40.91 ± 10.02 years), of which 20 with and 20 without a history of suicide attempts 22 HC (54.54% female, mean age 37.72 ± 13.63 years)	BD	MRI (1.5-T) BRAINS 2 software, SPM5	BD vs HC: Significantly smaller left cerebellum, right cerebellum and vermis. No volumetric differences between the BD subjects with and Without suicidal attempts.
Greenstein *et al*., 2011	Cross-sectional study	94 childhood-onset SCZ patients (47.25% female, mean age 17.25 ± 4.1 years) 80 related nonpsychotic siblings (53.75% female, mean age 16.88 ± 5.83 years) 110 HC (41.82% female, mean age 16.57 ± 4.44 years)	SCZ	MRI (1.5-T) BRAINS 2 software	SCZ vs HC: Smaller bilateral anterior lobes and anterior and total vermis volumes. Reduction in total, left, right, and bilateral posterior inferior cerebellum. No volumetric differences between siblings and HC, but differences in developmental trajectories of total and right cerebellum, left inferior posterior, left superior posterior, and superior vermis.
Roman-Urrestarazu *et al*., 2014	Cross-sectional study	60 subjects with FR (45% female, mean age 22.09 ± 0.7 years) 26 subjects with CR (65.38% female, mean age 22.08 ± 0.69 years) 13 subjects with FRCR (92.31% female, mean age 21.79 ± 0.7 years) 3 HC (58.90% female, mean age 21.96 ± years)	Psychosis risk group	MRI (1.5-T) FSL-VBM analysis	FRCR vs HC: lower GM volume in a cluster covering both cerebellar hemispheres and the vermis. FR had a cluster volume intermediate between HC and FRCR. Within FRCR there was an association between cerebellar cluster brain volume and motor function.
Wang *et al*., 2017	Cross-sectional study	18 first-episode SCZ-NCD patients (61.11% female, mean age 24.5 ± 6.70 years) 16 fist-episode SNZ-CD patients (31.25% female, mean age 22.63 ± 6.71 years) 21 HC (52.38% female, mean age 22.38 ± 3.94 years)	SCZ	MRI (3-T) SPM8	SCZ-CD vs SCZ-NCD: Reduced GM density in the vermis and tonsil of cerebellum, decreased GM density in the left supplementary motor area, bilateral precentral gyrus. Left supplementary motor area cluster could differentiate SCZ-CD from SCZ-NCD. Positive correlation between GM density values of the cerebellar vermis cluster and cognitive severity. SCZ-CD vs HC: Reduced GM density in the vermis and tonsil of cerebellum. GM density in cerebellar vermis tonsil cluster could differentiate SZ-CD from HC.
Segarra *et al*., 2008	Cross-sectional study	28 SCZ patients (25% female, aged 18–35 years) 28 HC (25% female)	SCZ	MRI (1.5-T) SPM2	SCZ vs HC: Lower GM volume in both cerebellar hemispheres and the vermis. Working memory dysfunctions in SCZ correlated with GM in both cerebellar hemispheres and vermis. Mental flexibility dysfunctions also correlated with reductions in WM volume in bilateral cerebellum.
Lupo *et al*., 2021	Cross-sectional study	29 BD patients (55.17% female, mean age 42.69 ± 10.53 years) 32 CD patients (43.75% female, mean age 46.81 ± 11.48 years)37 HC (59.46% female, mean age 45.75 ± 14.26 years)	BD	MRI (3-T) SPM8	BD vs HC: Clusters of significantly decreased GM volume that included right lobules I, IV and V, crus I, VIIB, IX and vermis crus II as well as left lobule VI and bilateral crus II BD and CD vs HC: Cerebellar alterations in GM that involved the anterior and posterior cerebellar regions. Pattern of overlapping GM loss in right lobule V, right crus I and bilateral crus II. BD vs CD: Significant difference in GM loss in cerebellar neurodegenerative patients in the bilateral anterior and posterior motor cerebellar regions, such as lobules I, IV, V, VI, VIIIa, VIIIb, IX, VIIb and vermis VI.

BD, bipolar disorder; CD, cerebellar neurodegenerative pathologies; CR, clinical risk but not family risk for psychosis; FR, family risk for psychosis; FRCR, both clinical and family risk for psychosis; GM, grey matter; HC, healthy controls; SCZ, schizophrenia; SCZ-CD, schizophrenia with cognitive deficits; SCZ-NCD, schizophrenia without cognitive deficits; T, tesla; VBM, voxel-based morphometry; WM, white matter.

## Results

Eighteen articles were included in the review. Of these, five articles were on patients with BD, 12 on patients with SCZ and one on subjects at psychotic risk. The study designs were cross-sectional for all studies, covering a time span from 2001 to 2021. As regards those studies that assessed patients with a diagnosis of SCZ, the comparison between psychotic patients and HC was carried out on heterogeneous populations, which varied for sample size, length of illness, onset period or clinical status. Either the total brain volume or the white matter and gray matter volumes of the cerebellar vermis were investigated, with the majority of the reviewed studies showing a reduction of total and gray matter volumes as well as an increase in white matter volumes.

In particular, the study by Ichimiya *et al.* (2001) highlighted significantly smaller volumes of the cerebellar vermis, but not of other cerebellar structures, in 20 drug-naive SCZ patients compared to 20 HC. Moreover, a correlation between the reduction in vermal volumes and specific subscales (depression and paranoia) of the Brief Psychiatric Rating Scale was highlighted (Ichimiya *et al*., 2001). Similarly, Loeber *et al*. (2001) confirmed the reduction of total vermis volumes in 38 SCZ patients compared to 26 HC. Indeed, the authors showed abnormalities in the posterior and anterior vermis areas but not in the inferior vermian level (Loeber *et al*., 2001). These results were further confirmed by three subsequent studies, which reported a reduction in total volumes of the cerebellar vermis in patients with chronic SCZ (Okugawa *et al*., 2003) and in patients with childhood-onset SCZ (Greenstein *et al*., 2011) and in adolescent patients diagnosed with SCZ (Henze *et al*., 2011) compared to HC. Also, a study by Wang *et al*. (2017) showed a reduction in gray matter density in cerebellar vermis in SCZ patients with or without cognitive impairments compared to HC.

Interestingly, Joyal *et al*. (2004) also reported selective reductions in the inferior vermis in 19 SCZ patients compared to 19 HC whereas Segarra *et al*. (2008) found a reduction in the total gray matter volume of the cerebellar vermis in adult patients compared to age- and gender-matched HC, respectively. Notably, cerebellar deficits have also been observed in subjects at psychotic risk in a study carried out by (Roman-Urrestarazu *et al.*, 2014). Indeed, the authors highlighted a cluster of lower gray matter at the cerebellar vermis level, in patients with both clinical and familiar risk of psychosis (Roman-Urrestarazu *et al.*, 2014) . Moreover, areas of specific reduction in gray matter volumes were identified in the whole superior vermis in 13 first-episode SCZ patients compared to 13 HC (Rasser *et al*., 2010) and only in the posterior superior vermis in 71 SCZ patients compared to 65 HC (Laywer *et al.*, 2006). Finally, Lee *et al*. (2007) highlighted an increase in cerebellar vermis white matter volume while the study by Lawyer *et al*. (2009) demonstrated an increased mean of gray matter/white matter ratios in cerebellar vermis in chronic SCZ patients with respect to HC.

With regard to BD patients, the results from the reviewed studies were highly heterogeneous. In particular, two studies seemed to highlight no differences between BD patients compared to HC (Brambilla *et al*., 2001; Monkul *et al*., 2008), with the former study showing, though, a reduction in total vermis volumes in family members of BD patients compared to HC whereas the latter highlighting a trend to smaller vermis V2 areas in BD patients compared to HC. Instead, two studies (Baldaçara et al., 2011; Lupo *et al*., 2021) found a significant reduction of cerebellar volumes in BD patients compared to HC without highlighting correlations with suicide attempts (Baldaçara et al., 2011). In contrast, the study by Womer *et al*. (2009) showed an increase in total vermis volumes in males but not in females affected by BD.

## Case presentation

When it comes to our observation, D.S. was a 35-year-old woman with a long history of psychiatric disorders with several past pharmacologic and psychotherapeutic treatments. Substantially normal psychomotor development was described, although her parents reported that she often stumbled as a child and was clumsy in her movements. She had a regular school background until she enrolled in university. Currently, she has been stuck in her university career for over 10 years, alternating between moments of great commitment and periods of intolerance and difficulty. The psychopathologic onset seemed to be around the age of 18 when, following family grief, the patient would have experienced an episode of depressive mood, with anhedonia and an increase in anxiety and brooding. Over the years, both the patient and family members have reported frequent mood swings in a depressive and hypomanic sense. In general, depressive experiences were accompanied by abandonment of studies, an intense sense of uselessness and defeat, as well as the need for continuous reassurance from family members. In particular, toward the mother, she undertook a relationship style of important emotional dependence, which in the depressive phases seemed particularly evident. In the hypomanic phases, D.S. had the feeling of being able to study, and appeared talkative, very casual, seductive, at times incongruous in the interaction.

When she was first admitted to our psychiatry ward, D.S. was going through a depressive phase, characterized by the fear of losing her reference figures. The anguish at the thought of separation from her mother, in the hypothesis of illness and death, which was not based on a concrete risk, triggered a solid suicidal ideation in her. The depressive thought, regarding the inevitable end of everything after the death of the mother, was not subjected to criticism and took on delusional characteristics. The thought of suicide seemed to be ruminative and stereotypical, not authentically connoted of a real planning. There were also anxious symptoms, such as the constant need for reassurance from the outside, as well as a certain infantilism in the behaviors. A therapy based on valproic acid 1000 mg, olanzapine 20 mg and clomipramine 25 mg per day was settled, to help the patient managing the suicidal rumination, the depressive symptoms and the delusional ideation. Thus, we assisted in the remission of the clinical picture (brief psychiatric rating scale from 44 to 27; Hamilton Rating Scale for Depression from 15 to 4, Young Mania Rating Scale from 0 to 9) (Hamilton, 1960; Overall and Gorham, 1962; Young *et al*., 1978).

**Fig. 2:**
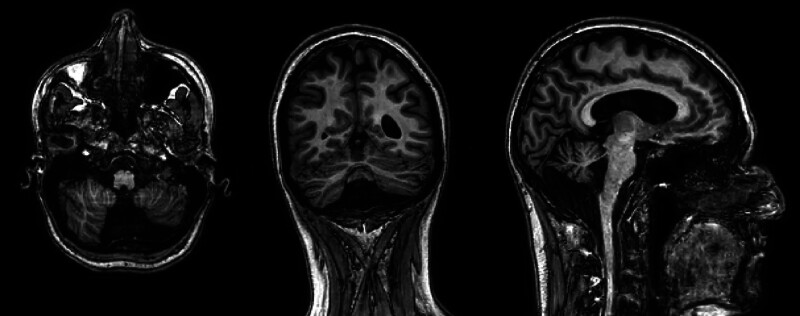
Structural MRI of the case presentation.

In the following months, we continued to witness D.S.’s mood swings, which profoundly influenced her planning skills in her study and life commitments. During the outpatient visits and in the two subsequent hospitalizations, psychometric assessment scales were carried out and the diagnosis of BD (via Structured Clinical Interview for Diagnostical and Statistical Manual of Mental Disorders—5° edition—Clinical Version) and of Dependent Personality Disorder (via Structured Clinical Interview for Diagnostical and Statistical Manual of Mental Disorders—5° edition—Personality Disorders) was formulated (APA, 2013; First *et al*., 2015a, 2015b). While depressive symptoms configured real depressive episodes, the counter-polar episodes seemed hypomanic. Frank delusional ideation did not manifest itself outside the acute phase of the disorder. In the context of the diagnostic assessment, neuroimaging evaluations were carried out. Brain MRI (Fig. 2) showed a large cisterna magna with hypoplasia of the inferior vermis and an accentuated cerebellar cortical pattern; ventricular size at upper limits of norm, minimal asymmetry of the lateral ventricles, midline structures in axis. On brain PET/computerized tomography, a picture of modest hypocaptation in the cerebellar vermis site was highlighted. At the specialist evaluation, there were no objective neurological alterations. A genetic evaluation was carried out in the suspicion of a syndromic picture, but no significant alterations were evidenced on the molecular cytogenetic analysis through an array of comparative genomic hybridization. On cognitive evaluations, the patient showed a normal IQ (92) (29.7% percentile), measured with the Raven’s Progressive Matrices (Bingham *et al*., 1966; SPM, 2008). On a brief neuropsychologic examination, the patient was found to be deficient (77/100) (5% percentile) in the global score (ENB-2) (Mondini *et al*., 2011), and in some cognitive domains, including abstraction, cognitive estimates, tangled figure test and clock test.

In light of the continuous fluctuations in D.S.’s mood, the poor therapeutic adherence and the ambivalent relationship with her mother, it was proposed to enter a rehabilitation residential program, which the patient accepted. After several therapeutic changes, D.S. found a partial thymic balance with the following therapy: valproic acid 1000 mg, aripiprazole 15 mg and fluvoxamine 50 mg daily. The choice to continue antipsychotic therapy is due on the one hand to the stabilization capacity of aripiprazole (Nestsiarovich *et al*., 2022), and on the other to the individual tendency towards clinical worsening in the face of attempts at pharmacological descalation. In recent months, she has been supported from the rehabilitation point of view with respect to the management of her daily life, seeking a mediation between excessive study commitments and surrender. Her social skills have also been strengthened to allow her a more functional social insertion. The patient was informed of the drafting of this case report and gave her consent to its publication.

## Discussion

The finding of an MRI picture of hypoplasia of the cerebellar vermis in the case of D.S. provided the opportunity for a review of the updated literature on cerebellar vermis alterations in subjects with psychotic disorders.

The cerebellum is a central brain structure with multiple functions, mainly motor coordination, cognitive and emotional control (Koziol *et al*., 2014; Adamaszek *et al*., 2017; Mariën and Borgatti, 2018; Schmahmann, 2019). It is physiologically connected to the spinal cord and the cerebrum; it has a complex regional organization, where the cerebellar vermis represents the medial cerebellar cortex (D’Angelo, 2018). Recent evidence is leading us to consider the cerebellum not only from the point of view of the motor regulation function but also from the behavioral and emotional perspective. Indeed, according to the hypothesis expressed by Schmahmann (2019), the cerebellum would implement the same regulatory function on both movement and behavior, thus maintaining an implicit homeostatic balance around the baseline (Schmahmann, 2019). In fact, according to Schmahmann (2019), the cerebellum maintains behavior around a homeostatic baseline, automatically, without conscious awareness. While the anterior regions are mainly involved in sensorimotor and vestibular control, the posterior regions are particularly involved in cognitive and emotional control (D’Angelo, 2018). In fact, a lesion in the posterior cerebellar lobe can give rise to a cerebellar cognitive affective syndrome, which is characterized by disorders in language, executive functions, visual processing and emotion regulation (Argyropoulos *et al*., 2020). The hypothesis of the emotion regulation function of the cerebellum has also been investigated from a psychopathological point of view: Minichino *et al*. (2014), in a review on cerebellar alterations associated with BD, express the theory of the cerebellum as an ‘emotional pacemaker’, therefore suggesting a role in emotional processing that would make the cerebellum an essential research target in affective disorders.

Notably, the majority of the reviewed studies reported the presence of structural abnormalities in cerebellar vermis in both SCZ and BD patients, selective with respect to the entire brain, with a mixed picture of either increased or decreased gray matter volumes in these patients compared to HC, which seemed to be maintained even in different types of patients (childhood-onset SCZ, adolescent or adult SCZ patients) and at a different stage of the course of illness (chronic psychosis, first-episode psychosis). Overall, these results would suggest an involvement of cerebellar alterations in psychotic disorders, as it is already documented for neurodevelopmental disorders. In fact, early cerebellar damage seems to impact the long-term outcome more than the late one, suggesting a function of the cerebellum in brain development (Stoodley, 2016; Sathyanesan *et al*., 2019). In line with this hypothesis is the evidence reporting the involvement of the cerebellum in the pathogenesis of autism spectrum disorders, attention-deficit hyperactivity disorder and psychotic disorders, which indeed further suggests the putative link between brain development and the emergence of these disorders (Phillips *et al*., 2015).

Many studies have demonstrated that patients affected by SCZ or BD show neurodevelopmental abnormalities, which seem to accumulate from the prenatal period to adolescence, constituting a biological vulnerability to developing the disorders (Rund, (2018)). Therefore, since cerebellar abnormalities are often related to neurodevelopmental problems, their presence in psychotic patients seems to confirm the hypothesis of SCZ as a neurodevelopmental disorder.

Furthermore, in recent years, many theories have proposed the key role of the cerebellum in emotion regulation (i.e. emotional pacemaker theory) (Heath, 1977; Minichino *et al*., 2014), it is not surprising that many studies on affective disorders found selective deficits in this region (Adamaszek *et al*., 2017). Also, it might be possible that structural abnormalities in this structure have a role in the pathogenesis and in the progress of psychotic disorders, whose psychopathologic features are characterized by the difficulty of recognizing internal and external stimuli and creating a valid construct of reality.

Nevertheless, it is important to highlight that the reviewed studies suffer from some limitations. First, the heterogeneity, in terms of illness severity, course of illness (first-episode psychosis vs. chronic SCZ) and medication status (drug-naïve patients vs. patients in treatment with antipsychotic drugs), of the sample employed might have negatively impacted the generalizability of the findings. Second, the reviewed studies used MRI scanners with different magnetic fields (1.5T vs. 3T), which could impact the uniformity of the results. Finally, all studies included are cross-sectional, which limits the possibility of longitudinal evaluations of the results.

In conclusion, while it is possible to hypothesize an association between cerebellar vermis hypoplasia and SCZ, further studies are needed to define this association in the context of BD. Interestingly, this case report highlights how, in a case of BD with psychotic symptoms, there was an incidental finding of hypoplasia of the cerebellar vermis, not associated with neurological symptoms. It would be interesting to understand if cerebellar vermis abnormalities could be more frequently related not only to SCZ but also to other psychotic disorders, such as BD. Although further studies are needed to fully understand the relationship between cerebellar vermis hypoplasia and psychotic disorders, the interesting finding of this case report could provide a link between neuroimaging research and clinical practice. Indeed, even if the clinical use of neuroimaging tools has still a not decisive impact as an aid in the formulation of the diagnosis, the study of individual cases in which brain abnormalities are highlighted can provide a solid foundation for the promising developments of treatment personalization (Aydin *et al*., 2019; Keshavan *et al*., 2020).

## Acknowledgement

This study was (partially) supported by the Italian Ministry of Health (Ricerca Corrente 2023) and by the Italian Ministry of Health (Grant No. GR-2019-12369100).

### Conflicts of interest

There are no conflicts of interest.
